# Case of Delivery per Anum

**Published:** 1839-03-16

**Authors:** John Andrews

**Affiliations:** Steubenville, (Ohio)


*Case of Delivery per Anum.* By JOHN ANDREWS, M. D.

A case of delivery per anum occurred in this place, about two years ago, in the hands of a midwife who then had considerable practice. The midwife stated to me that she was sitting by the fire, when the woman called to her for assistance, and that on examining, she found the head of the child "coming the wrong way." The child was of full size, and was delivered in a few minutes completely *per anum.* The perineum was torn about an inch, but not directly towards the fourchette, and thereby a *complete* division between the rectum and vagina was avoided. The bowels of the patient were kept constipated for a number of days, and thus a perfect reunion of the laceration effected. It was the first child. It may not be deemed out of place here, to remark that he who has had but a limited experience in obstetrics, will have met with cases, in which the necessity was urgent, to support the vagino-rectal septum, in order to avoid its rupture by the head of the child. This important point in obstetrical practice is seldom indicated in systematic works. I feel quite confident that I have met with several cases in which, but for a strong and well sustained support to the anus, this terrible accident must have occurred. There is a decided difference between supporting the *perineum*, and recto-vaginal septum; and the distinction is of the greater importance, inasmuch as pressure upon the former, at the moment when the latter is threatened, must tend greatly to increase the danger. The head should not be obstructed, in its passage over the perineum, until all risk of diverting from its proper course has passed; on the contrary, pressure posterior to the perineum properly directed, tends not only to prevent a rupture into the rectum, but also to advance the head forward through the vulva.

*Steubenville*, (*Ohio*,) March 9, 1839.

